# Casual Dock Work: Profile of Diseases and Injuries and Perception of Influence on Health

**DOI:** 10.3390/ijerph110202077

**Published:** 2014-02-19

**Authors:** Marta Regina Cezar-Vaz, Marlise Capa Verde de Almeida, Clarice Alves Bonow, Laurelize Pereira Rocha, Anelise Miritz Borges, Diéssica Roggia Piexak

**Affiliations:** 1 School of Nursing, Federal University of Rio Grande, Rio Grande, RS 96203-900, Brazil; E-Mails: marlisealmeida@msn.com (M.C.V.A.); laurelize@gmail.com (L.P.R.); miritzenfermeira@yahoo.com.br (A.M.B.); diessicap@yahoo.com.br (D.R.P.); 2 Federal University of Pampa, Uruguaiana, RS 97500-970, Brazil; E-Mail: claricebonow@unipampa.edu.br

**Keywords:** occupational health, occupational diseases, accident prevention

## Abstract

The present study aimed to identify the profile of diseases and injuries that affect casual dock workers and identify casual dock workers’ perceptions of positive and negative work influences on their health. This study consisted of two phases. The first phase was a quantitative study composed of a retrospective analysis, conducted with 953 medical records. The second phase of the research is a non-random sample with 51 casual dock workers. Data analysis was performed with SPSS 19.0. The average age of the casual dock workers was 48.7. Concerning working time, the majority had more than 19.6 years of dock work experience. In the first phase, 527 pathologic diagnoses were identified. The diagnoses that affected the musculoskeletal system (15.8%, *N* = 152; *p* < 0.01) were highlighted. Consequences to physical health produced by accidents stood out, with fracture registration predominating (12.8%, *N* = 122; *p* < 0.05). Significant differences were found for positive work influence on the cardiovascular system and family health. It was concluded that the diagnoses obtained are related to the influence of dock work perception and have motivated an introduction of preventive measures.

## 1. Introduction

Ports are important working environments for the economy and the world markets [1]. In Brazil, the ports are the main entry and exit for trade with other nations. According to the Ministry of Development, Industry and Foreign Trade, in 2011, Brazilian ports were responsible for 95.9% of exports and 88.7% of imports into the country [2]. Through its ports Brazil is one of the main suppliers of raw materials in the World, and its trade relations with the outside world have been increasing. In 2011, over 80% of the Brazilian trade passed through the country’s ports, which amounted to about 653 billion tons of transported goods with an estimated value of U.S. $387 billion. South Brazil is currently seeing a growth in shipping activities, as the Seaport of Rio Grande which handles the highest volume of exported products, totaling around 480,000 tons in 2012 [2], is located in this region.

On the other hand, port working conditions can produce health problems and disorders for the workforce. Workers need to be safe in their work environment to maintain their well-being and to remain active. A healthy worker generates greater productivity and sustainability for local and national companies [1]. The assistance service for casual dock workers (CDWs) contributes towards preventing work-related diseases and occupational accidents beyond clinical knowledge. Studying the information provided by this service helps to visualize the health of the CDWs, a population still poorly investigated.

The International Labour Organization (ILO) estimates that of the total of 2.34 million work-related deaths per year, around 321,000 (14%) are related to accidents, while others are due to work-related diseases [3]. Studies in port environments have highlighted the possible health consequences of this activity. The European Union, North America, Japan and Australia stand out in these statistics; the main reasons for the occurrence of occupational injuries are leaks, fires, explosions and poisoning by gas [4]. In the United States and United Kingdom, accidents occur on board the vessels due to the handling of improperly packed hazardous materials. Accidents caused a significant number of deaths between the years 1998 and 2008 [5]. The mode of storing materials also produces risks, for example, working with containers. The study ascertained that the use of this equipment has improved productivity and cargo handling by workers, although this has not been reflected in health and safety improvements as the number of accidents related to cargo handling have increased [6].

Brazil has approximately 19,177 registered CDWs, subdivided into 24 organized ports [7]. These workers are characterized by their supply of services to various companies, without employment through mandatory mediation, coordinated by Órgão Gestor de Mão-de-Obra (OGMO) [8]. In Brazil, the work is divided into six occupational categories: terminal handlers, stevedores, public weight masters, vessel guards and maintenance workers [8]. These workers work on automated loading or unloading (bulk liquid) of vessels, handling of solid bulk, ground and airborne cargo, moving spare parts and supplies and craft supplies, among others. They also perform the confirmation, manipulation, storage, metering and delivery of cargo, notes relating to the characteristics, origin or destination of the products, as well as checking and repairing the commodities [8].

Studies highlight maritime industry workers’ risk perception and accidents, which, in Brazil are related to the worker falling to the ground or the sea, falling objects which have been suspended, exposure to noise and weather [9], heavy loads to be moved [10], and in Norway, crushing by machine parts, slippery surfaces, exposure to explosions, blow-out, fire, noxious gases and sabotage [11,12]. Besides the accidents, occupational diseases self-reported by maritime industry workers of a port in southeastern Brazil are also highlighted, which affected the regions of the spine and joints [10], ergonomic problems in Sweden [13] and quality of sleep in a port in Norway [14]. Another study has also identified the occurrence of mental alterations caused by disorders of the sleep-wake cycle and the mental strain related to port working conditions [15]. In addition to these studies, in a sample of 306 CDWs of a port in southern Brazil, 71.89% reported having had disturbances in the musculoskeletal system and 41.5% reported mental disorders [16].

Overall, occupational diseases are a source of extreme suffering and absenteeism in the workplace. However, although occupational diseases are annually responsible for killing six times more people than work-related accidents, diseases remain invisible. Furthermore, the nature of these diseases changes rapidly because of the technological and social changes, combined with the global economic conditions, aggravating existing health hazards and generating new risk factors [17]. The record of these diseases is particularly important against the changing patterns of work and technology. The recognition that a disease has an occupational origin (in whole or in part) influences the choice of devices used in medical surveillance and enhances awareness of appropriate preventive measures [3].

For these reasons, the present study aimed to identify the profile of diseases and injuries that affect casual dock workers and identify casual dock workers’ perception of positive and negative work influence on their health. As specific objectives, the aim has been to: identify work-related diseases diagnosed in a Medical Outpatient Clinic for Dock Work; identify work-related injuries recorded at a Medical Outpatient Clinic for Dock Work and evaluate the casual dock workers’ perception about the positive and negative work influence to health.

## 2. Methods

This study consists of two phases. The first phase is a quantitative, descriptive study with retrospective analysis. Data sources were 953 medical care files of the Medical Outpatient Clinic for Dock Work. The study was conducted in 2011 in Rio Grande, Rio Grande do Sul, Brazil. The second phase consists of an exploratory and descriptive pilot study with CDWs, conducted in 2012, in the same county.

This study is part of a larger research project entitled “Health, Risks and Occupational Diseases: An Integrated Study in Different Work Environments” [18]. It was approved by the Research Ethics Committee of the Federal University of Rio Grande (Universidade Federal do Rio Grande—FURG). CDWs were included in the study after signing an informed consent form. The study was conducted using public funds (National Counsel of Technological and Scientific Development—CNPq) and was linked to the research group Laboratory of Socioenvironmental Process Studies and Collective Production of Health (LAMSA) of the Nursing School at the Federal University of Rio Grande.

Occupational disease is understood here as any disease contracted as a result of exposure to risk factors arising from work. It presents causal relationship with exposure to a particular work environment or work activity that produces certain specific diseases. Work-related diseases may have multiple causes, in which the factors of the working environment act as triggers or enhancers, along with other risk factors for the development of such diseases [19]. Occupational accidents are all unexpected and unforeseen, including acts of violence, derived from work or related to it, which result in injuries, illness or death of one or more workers [20].

### 2.1. Subjects

In the first retrospective phase, a data source of 953 medical care files from the Medical Outpatient Clinic for Dock Work, affiliated with the *Órgão Gestor de Mão de Obra do Trabalho Portuário Avulso do Porto Organizado do Rio Grande* (OGMO-RG) was used. In the second prospective phase, a non-random convenience sample of 51 CDWs was selected. The following inclusion criteria were: be a CDW and participate in the activities of the Prevention of Accidents in Port Work Week (SIPATP).

SIPATP is a specific convention of OGMO to discuss different topics concerning CDWs’ health. It presents various activities to educate workers about accident prevention. In this port workweek, the moments in which the CDWs were assembled were used to conduct the questionnaire about their perception of positive and negative influences of work on their health. This sample is not population-representative because the respondents were only CDWs who participated in the activities of SIPATP.

In Brazil, CDWs is performed in six categories [8]: terminal handlers: moving goods activity—receiving, checking, internal transport, opening packages, among others; stevedores: goods movement on the decks or in the holds of vessels, when performed with equipment on board; public weight masters: counting volumes and annotation of their characteristics, origin or destination, check condition of goods, weighing assistance, among others; vessel guards: monitoring entry and exit of persons on board vessels moored or anchored in port; maintenance workers: repair and restoration of goods packaging, loading and unloading of vessels; and workers block: cleanup activities and preservation of merchant vessels and their tanks. For this study, the maintenance workers and workers block were grouped together due to the similarities of the work carried out by these categories and the small number of workers in each category.

### 2.2. Data Collection

In the first phase, which was retrospective, the secondary data collection was performed by a pre-form compound with the following variables: participant characteristics (gender, professional category, age, skin colour and working time in the sector) and; clinical diagnoses classified according to the chapters of the International Classification of Diseases 10 (ICD-10) [21,22], as follows: Certain infectious and parasitic diseases (A00-B99); Diseases of the blood and blood-forming organs and certain disorders involving the immune mechanism (D50-D89); Endocrine, nutritional and metabolic disease (E00-E90); Mental and behavioural disorders (F00-F99); Diseases of the eye and adnexa (H00-H59); Diseases of the ear and mastoid process (H60-H95); Diseases of the circulatory system (I00-I99); Disease of the respiratory system (J00-J99); Diseases of the musculoskeletal system and connective tissue (M00-M99) and the harms arising from dock work (traumas, amputations, cuts, rupture of tendon, fissured fracture, crushing injuries, prehension, falls, fractures, strains, sprains, dislocations and animal bites) [19]. ICD-10 was used because the assistance service uses this to classify diseases. Data collection was performed in 2010. Clinical records collected comprise the period from 2000 to 2009.

For the second phase, a self-administered questionnaire was used to collect self-reported data. The questionnaire addresses CDWs’ perception of positive and negative work influences on their health. Each worker was individually asked to assign positive scores (0 to 10) and negative (0 to −10) for the influence of the work process on their health. At this phase, data collection was performed during SIPATP in 2012.

The first phase of the questionnaire was validated by LAMSA and its reliability was assessed by Cronbach Alpha test, obtaining the result of 0.945, demonstrating that the variance of the score of the issues is understandable in terms of the second phase questionnaire.

### 2.3. Data Analysis

For the study data analysis of the first phase, descriptive analysis was carried out first and further inferential analysis was then performed. Chi-squares were used to test the hypothesis of association/dependence between the two variables, from the sets of the proportions observed. Besides this analysis, adjusted analysis was also performed, which should present a value greater than or equal to 1.96 to be statistically significant. *α* = 0.05 were considered significant.

For the second phase of the data analysis, the normality of the distribution was examined by the Kolmogorov-Smirnov test. Following this, Student t test was performed to evaluate the work influence on health. *p*-values of <0.05 were considered significant. All data analyses were conducted with SPSS software, version 19.0.

## 3. Results

### 3.1. Participant Characteristics

The results include records of 953 medical care files of CDWs. The CDWs were all male. Most records, 523 (54.9%) were terminal handlers; 309 (32.4%) stevedores; 66 (6.9%) public weight masters; 28 (2.9%) vessel guards and; 25 (2.6%) maintenance workers. Two medical files did not indicate the worker’s professional category (0.2%). Other data obtained from the medical records can be viewed in Table 1.

### 3.2. Diagnosis in Casual Dock Workers

527 diagnoses were identified [18]. Four disease groups have a higher frequency of cases, which are: diseases of the musculoskeletal system and connective tissue, in which 170 diagnoses were detected, in which fifteen workers had more than one pathological diagnosis, totaling 152 affected workers (15.8%); diseases of the circulatory system (9.1%, *N* = 87); diseases of the respiratory system (2.6%, *N* = 25 and mental and behavioural disorders (2.2%, *N* = 17). In Table 2 the number of diagnosis and their respective qualifying groups as ICD 10 [18] is presented.

**Table 1:** Demographic characteristics of the casual dock workers obtained from medical care files (*N* = 953).

Professional Category	*N*	%
Terminal handlers	523	54.9
Stevedores	309	32.4
Public weight masters	66	6.9
Vessel guards	25	2.6
Maintenance workers	28	2.9
**Age (years)**		
<50	496	52
>50	457	48
**Working time**		
<19	493	51.7
>19	460	48.3
**Skin color/ethnicity**		
White	757	79.4
Black	143	15
Asian	4	0.4
Brown	38	4.0
Indigenous	2	0.2

There were other less prevalent diseases, such as leptospirosis, which affected one (0.1%) terminal handler; one (0.1%) case of leukopenia in a public weight master; four (0.4%) cases of mental disorders, not specified—two (0.2%) cases among stevedores and two (0.2%) cases among terminal handlers; carpal tunnel syndrome occurred with one (0.1%) terminal handler; three cases of viral conjunctivitis occurred with two (0 2%) terminal handlers and one (0.1%) stevedore; five cases of acute myocardial infarction, occurred with two (0.2%) terminal handlers, two (0.2%) stevedores and one (0.1%) public weight master.

There were also two cases of acute sinusitis, one (0.1%) stevedore and one (0.1%) public weight master; three (0.3%) terminal handlers were affected by dermatitis; three cases of arthropathy occurred with terminal handlers (0.1%) and stevedores (0.2%); seven cases of enteropathy, unspecified, which affected two (0.2%) terminal handlers and five (0.5%) stevedores; a case of trigger finger, which affected a stevedore (0.1%) and three cases of synovitis and tenosynovitis, affecting one (0.1%) terminal handler and two (0.2%) stevedores.

There is a significant association between the variable “professional category” and the variables: leukemia (*p* < 0.05), diabetes (*p* < 0.01), tendinitis (*p* < 0.01); corrective lenses (*p* < 0.01); essential hypertension (*p* < 0.01), lumbuciatalgia (*p* < 0.05) and other cardiac arrhythmias (*p* < 0.01). Multivariate analysis facilitated visualization of the association between leukemia, corrective lenses, essential hypertension and arrhythmia, which was significantly higher for public weight masters. 

**Table 2:** Diagnosis in casual dock workers according to ICD-10, absolute number and percentage.

		Certain infectious and parasitic diseases		Diseases of the blood and blood-forming organs		Endocrine, nutritional and metabolic diseases			Mental and behavioural disorders			Diseases of the eye	Diseases of the ear and mastoid process		Diseases of the circulatory system		Diseases of the respiratory system		Diseases of the musculoskeletal system and connective tissue							
		Tuberculosis	Hepatitis	Leukemia *	Nutritional anaemias	Diabetes **	Hypercholesterolaemia	Obesity	Depressive episode, unspecified	PD	RSS	PSCL **	OHL	Labyrinthitis	EPH **	OCA **	ARU	CLRD	Low back pain	Lumbociatalgia *	Cervicobrachial syndrome	Dorsalgia	Arthrosis	Artralgia	OEUT **	Other enthesopathies
**Terminal handlers**	N	5	6	0	1	5	20	20	5	1	1	9	20	2	33	0	2	7	32	3	9	2	10	1	16	7
	%	0.5	0.6	0	0.1	0.5	2.1	2.1	0.5	0.1	0.1	0.9	2.1	0.2	3.5	0	0.2	0.7	3.4	0.3	0.9	0.2	1.0	0.1	1.7	0.7
**Stevedores**	N	5	10	0	2	19	15	26	3	0	2	30	8	3	19	1	3	4	20	4	7	3	4	2	6	5
	%	0.5	1.0	0	0.2	2.0^(^^+)^	1.6	2.7	0.3	0	0.2	3.1	0.8	0.3	2.0	0.1	0.3	0.4	2.1	0.4	0.7	0.3	0.4	0.2	0.6	0.5
**Public weight masters**	N	0	3	1	1	2	1	3	3	0	2	2	2	0	20	2	1	3	5	3	0	0	0	1	1	1
	%	0	0.3	0.1^(^^+)^	0.1	0.2	0.1	0.3	0.3	0	0.2	0.2^(^^+)^	0.2	0	2.1^(^^+)^	0.2^(^^+)^	0.1	0.3	0.5	0.3	0	0	0	0.1	0.1	0.1
**Maintenance workers**	N	0	0	0	0	2	1	3	0	0	0	0	0	0	3	0	1	1	1	1	1	0	1	0	0	0
	%	0	0	0	0	0.2	0.1	0.3	0	0	0	0	0	0	0.3	0	0.1	0.1	0.1	0.1	0.1	0	0.1	0	0	0
**Vessel guards**	N	0	0	0	0	1	1	1	0	0	0	0	0	0	4	0	0	1	1	0	2	0	0	0	7	0
	%	0	0	0	0	0.1	0.1	0.1	0	0	0	0	0	0	0.4	0	0	0,1	0.1	0	0.2	0	0	0	0.7^(^^+)^	0

**Notes:** *p*-value: * *p* < 0.05; ** *p* < 0.01. PD—Panic disorder (episodic paroxysmal anxiety); RSS—Reaction to severe stress and adjustment disorders; PSCL—Presence of spectacles and contact lenses; OHL—Other hearing loss; EP—Essential (primary) hypertension; OCA—Other cardiac arrhythmias. ARU—Allergic rhinitis, unspecified; CLRD—Chronic lower respiratory diseases; OEUT—Other Enthesopathy, unspecified—Tendinitis NOS; ST—Synovitis and tenosynovitis. ^(+)^ Significant association between two categories/Analysis waste.

The occurrence of diabetes was significantly higher for stevedores. The occurrence of tendinitis showed greater significance among vessel guards.

Adjusted analysis using the professional category also showed a higher significance between the non-occurrence of diagnoses among CDWs, highlighting the lack of diagnosis of leukemia, diabetes, use of corrective lenses, essential hypertension and arrhythmia in terminal handlers.

### 3.3. Injuries for Casual Dock Workers

The results concerning the injuries produced by accidents, obtained from the medical records, are presented in Table 3 as “Occupational Injury and Illness Classification Manual” [19].

**Table 3:** Injuries for casual dock workers, absolute number and percentage.

Variables	Terminal Handlers	Stevedores	Public Weight Masters	Maintenance Workers	Vessel Guards
Traumas	10	5	0	0	0
	1.0%	0.5%			
Amputation	1	2	0	0	0
	0.1%	0.2%			
Cut/abrasions/injuries	24	15	4	1	2
	2.5%	1.6%	0.4%	0.1%	0.2%
Rupture of tendon	6	2	0	0	0
	0.6%	0.2%			
Fissured fracture	4	1	0	0	0
	0.4%	0.1%			
Crushing injuries	2	0	0	0	0
	0.2%				
Prehension	2	0	0	0	0
	0.2%				
Falls, slips, trips	12	4	1	1	4 *
	1.3%	0.4%	0.1%	0.1%	0.4%
Fractures	75	44	1 **	1	1
	7.9%	4.6%	0.1%	0.1%	0.1%
Bruises, contusions	74	36	4	1	4
	7.7%	3.8%	0.4%	0.1%	0.4%
Sprain	32	30	4	1	3
	3.4%	3.1%	0.4%	0.1%	0.3%
Dislocations	8	3	0	0	0
	0.8%	0.3%			
Bitten by animals *	4	1	0	0	0
	0.4%	0.1%			

Notes: * *p* < 0.01; ** *p* < 0.05.

The analysis showed significant association between the variable “professional category” and the occurrence of fractures (*p* < 0.05) and falls (*p* < 0.01). Multivariate analysis showed that there was a greater association between falls and vessel guards and non-occurrence of fractures among public weight masters.

### 3.4. Perception of the Work Influence on the Health of Casual Dock Workers

In the second phase of the study, the participation of 51 CDWs was obtained. All were male, 37 (72.5%) terminal handlers, seven (13.7%) stevedores, three vessel guards (5.9%) and two (2%) maintenance workers. There was one participant who did not mention the professional category to which he belonged. The average age of the participants was 49.2 years and working time in the port was 26.1 years. Regarding lifestyle, 31 CDWs reported being smokers (60.8%), 26 reported not drinking alcohol (51%), 31 felt they ate properly (60.8%) and 25 performed regular exercise (49%).

Table 4 presents the results of the means, standard deviation (SD) and p-value related to the positive and negative CDW work influence. The CDWs identified positive influence of work on the cardiovascular (*p* = 0.002), gastric and mental system, on physical activities and family health (*p* = 0.009). CDWs identified negative influence of the work for the respiratory, integumentary, ocular, auditory, musculoskeletal systems, for sleep and maintenance of dietary habits. There was no significant difference in the mean values for each variable studied.

**Table 4:** Positive or negative influence from work on CDWs’ health (*n* = 51).

Variables	Mean	SD	*p-value*
Cardiovascular system	2.18	4.75	0.002
Respiratory system	−0.98	6.60	0.294
Integumentary system	−0.16	5.25	0.832
Gastric system	0.20	5.43	0.798
Ocular system	−0.73	5.45	0.347
Auditory system	−1.35	5.29	0.074
Mental system	1.06	6.37	0.241
Musculoskeletal system	−0.98	5.98	0.268
Sleep	−0.68	5.88	0.432
Dietary habits	−0.02	6.41	0.982
Physical activity	1.31	5.70	0.116
Family health	2.08	5.35	0.009

## 4. Discussion

Work-related diseases and injuries are a public health problem in Brazil and worldwide. The importance of collecting health information to strengthen health surveillance of workers is emphasized. The characteristics of the port work and CDWs have aspects that are important to establish action strategies for the prevention of these diseases and injuries. For example, the CDW study participants were all men with a mean age 50, which may indicate greater vulnerability to the development of chronic diseases [23].

Technological changes, both social and organizational, produce emerging risks. As an example, inadequate ergonomic conditions, such as shift work, repetitive activities, mechanical vibration, bad posture, among others can be cited. These ergonomic conditions can trigger muscle disorders, the diversified incidence of which were prominent for CDWs in this and other studies [10,16]. The development of these diseases strengthens the negative influence on the musculoskeletal system identified by the CDWs. In Brazil, the pathologies and muscular changes occur more often among younger workers. This occurrence reinforces the possible determination of working conditions [24].

The data showed a significant occurrence of chronic diseases, such as diabetes, use of corrective lenses and arrhythmias. Among the categories of CDWs, public weight masters, who had a higher mean age (60.9 years), were shown to be a profession greatly affected by these conditions. Diabetes becomes more common among older individuals, for example, according to a Brazilian epidemiological study. The southern region of Brazil, location of the development of this study, has the second highest prevalent disease diagnosis, and this confirms the importance of data on health care for port workers [25].

Another important risk identified and discussed by the ILO is psychosocial. This risk is due to the organization and management of work due to boredom or excessive work rate, productivity requirements, labour relations conflicts and gaps in training for work requirements of supervision of workers, among others. Such risks produce diseases as mental disorders and lead to risky behaviour of workers. Within this behaviour ranking is the abuse of alcohol and drugs [3], which has been identified in the same port in which this study was conducted [26].

Mental disorders, such as stress, are also related to cardiovascular diseases [3]. In this study, the cardiovascular system diseases are the most frequent pathologies. In a previous study, at the same port [16], the cardiovascular diseases were highlighted by the dock workers. These results might be related to the physical effort demanded at work, as it has been identified with workers from a casting company [27] and among truck drivers [28]. Besides these, another study presented a relationship between cardiovascular diseases and physical effort in three Eastern Asia countries [29].

On the other hand, the work carried out at the port, enable the CDWs to remain physically active, reducing the risk of developing cardiovascular diseases and other chronic ones, such as diabetes and hypertension [30]. Thus, it should be considered that the port work may have a positive influence on the CDWs’ health, as although cardiovascular diseases have been frequent in the population studied, the fact that the port activities involve physical activity may have facilitated the identification of a positive influence of work on the cardiovascular system.

Requesting and analysing periodic health exams and educating CDWs towards a healthier lifestyle helps preventing work-related illnesses. These activities increase the risk perception of workers about the events that surround the cardiovascular system [28,29].

Shift work is once again remembered as it regards questions related to the sleep-wake cycle, which is also negatively influenced by port work. The rate of charge and discharge in Brazilian ports involves shifts of day and night work and this interferes with the sleep quality of workers. With the definition of at least 11 consecutive hours of rest every two periods of work, it is possible to restore the regularity of the sleep-wake cycle. According to the collective bargaining agreements between OGMOs and CDWs unions this can establish different work routines. This is what happens at the port where the study was developed because terminal handlers, for example, work a minimum of 6 hours, justified by the interest of both parties in this cycle [30].

It is important to analyze the cost-benefit ratio. The decrease in the rest interval provides financial benefits to CDWs; however, the absence of the sleep period causes long working hours, insufficient sleep and fatigue. This will increase the risk of accidents, so it is necessary to implement measures to reduce fatigue and improve sleep quality [31].

Asthma and bronchitis are chronic respiratory diseases which occur more frequently among the CDWs. This occurrence may be associated with genetic and personal factors of the workers as much as working with the transport and handling of different loads and products. This work exposes the CDWs to physical hazards, like dust, which triggers reactions such as acute respiratory nasal irritation, cough, and expectoration to the development of chronic changes, which may be related to long-term exposure. Soya beans, for example, in their different forms (bran, husk and soybean), moved in large quantities at the port where the study was conducted, contain a protein capable of triggering allergic processes, which provoke respiratory symptoms or diseases [32].

Regarding the influence of work on the auditory system, the CDWs identified a negative influence of work for hearing. It is highlighted that the port environment has been nominated as noisy by CDWs [9] in another study conducted in the same port. The port characteristics include the noise of the vessels and work within them, which increases the magnitude of the sound and generates disturbances affecting, not only hearing but different organ systems of the body, as for example, the mental system.

Another important aspect is the positive influence of work on mental health. This can be justified because the CDWs identified as a positive factor the possibility of diversifying shifts. However, the study found that the mental well-being and physical labour is not only associated with the workload and working time but also if the working hours coincide with the workers’ preferences for working hours [33].

Furthermore, the average of the positive influence of work on mental health was not significant and this can be justified because the routine work in the port environment is very intense; the worker may be obliged (or prefer) to diversify their shifts of operation. This contributes to the occurrence of health damage related to stress and depression and instability in family relationships [33].

In this sense, the work can be considered as positive for family health. The study shows that there is a decrease in worker-family interaction as a result of the work routine [34]; however, among the CDWs, we have the consideration that the diversity of work periods and financial issues justify the positive influence of work on mental health. The CDW is often the sole provider, which indicates their work as being positive as it allows for the financial investment required to purchase goods and services necessary for the life of the family (not necessarily for family life).

The occurrence of injuries affecting health, which is also capable of producing changes in the routine of workers, reflects body constraints which lead to forced interruption of the work and leading to the development of health problems resulting from their occupation is highlighted.

Fractures produced by occupational accidents are considered serious injuries that cause immobilization of the affected limb. They can cause muscle atrophy, decreased extensibility and decreased range of motion [35], losses which directly interfere in workers’ mobility, delaying their return to work and jeopardizing their lives. Fractures can produce permanent incapacity to work or even be fatal, due to the possibility of injury to vital organs located in the thorax, spine and head.

The occurrences of fractures had already been reported among workers of the oil industry [36] and other associated areas [37], and in the present study were significant in the category, CDWs vessel guards, which operate in monitoring the entry and exit of people and goods on board ramps, basements and decks, among other embarkation places. Unlike the findings presented, studies addressing the health worker present a vigilant focus on cardiovascular health [38] and the influence of shift work on health [39], indicating the occurrence of injuries related to accidents as an important field for surveillance and health protection of workers.

Another example of the result of a significant impact on the port environment was bruises and injury/injuries related to work performance. The most affected categories were stevedores and terminal handlers, whose activities are similar. The work involves a certain level of vulnerability and involves potentially harmful exposure, as they use manual and mechanized tools capable of producing lesions, which might justify the pathological occurrence.

Health surveillance of workers in relation to occupational accidents is lead primarily by prevention and the use of personal protective device (PPD). In this sense, it is necessary to prioritize worker’s comfort in the use of equipment, considering that the individual often works in unhealthy environments, under extreme temperatures, confined spaces and difficult access, among others, which causes discomfort and consequent failure to use the equipment to facilitate job performance. Moreover, not every result produced by routine work is prevented by the use of such equipment, which justifies the need for monitoring services for the enhancement of self-care with the expansion of risk perception present in ports.

Considering the result of this academic study, on the diseases and harms, as well as on the influence of work in the health of the small group of workers, the implication of this study leads to proposing the organization of a health interdisciplinary service, which includes the participation of the port workers’ union in order to propose preventive measures for diseases and harms and which can promote health.

## 5. Conclusions

In conclusion, CDWs present several diseases and harms that may be related to work. The most frequent were the diseases of the musculoskeletal system and the connective tissue, blood system diseases and the diseases of the endocrine, nutritional and metabolic systems. Such data added to the frequency of hazards and the perception of the negative influence of the work for most of the factors which have been questioned enable us to state that the dock work environment is particularly dangerous. However, the perception of the influence of the work for the blood system and for the family health (statistically significant) shows the positive influence of the physical effort at work and the possibility to support the family.

Knowledge of the diseases and injuries that affect CDWs and identify CDWs’ perception of positive and negative work influence on their health can be acquired by providing healthcare professionals with subsidies for the prevention of situations which favour their occurrence in relation to environmental aspects.

Thus, it is necessary to introduce a greater number of intervening environmental actions to ratify the importance of building subsidized clinics to improve the quality of health surveillance, in the reorganization of the work process and the delimitation of technological tools capable of influencing workers’ behaviour, thereby increasing the positive influence of work for CDWs.

Study limitations were identified, such as the focus on the presentation of the consequences produced by possible occupational accidents and not in the situations of accident *in loco*. The data source possesses limits by the very characteristic of the service because it is a service centered on port workers and often cannot include specifications about the disease. However, it has been identified that the study has relevance for making public, in a scientific manner, a profile of a pathological group of workers. Based on this knowledge, investments in planning for future health surveillance in the workplace of ports are recommended.
